# Nonvolatile programmable silicon photonics using an ultralow-loss Sb_2_Se_3_ phase change material

**DOI:** 10.1126/sciadv.abg3500

**Published:** 2021-06-16

**Authors:** Matthew Delaney, Ioannis Zeimpekis, Han Du, Xingzhao Yan, Mehdi Banakar, David J. Thomson, Daniel W. Hewak, Otto L. Muskens

**Affiliations:** 1 Zepler Institute, Faculty of Engineering and Physical Sciences, University of Southampton, SO17 1BJ Southampton, UK.; 2 Physics and Astronomy, Faculty of Engineering and Physical Sciences, University of Southampton, SO17 1BJ Southampton, UK.

## Abstract

The next generation of silicon-based photonic processors and neural and quantum networks need to be adaptable, reconfigurable, and programmable. Phase change technology offers proven nonvolatile electronic programmability; however, the materials used to date have shown prohibitively high optical losses, which are incompatible with integrated photonic platforms. Here, we demonstrate the capability of the previously unexplored material Sb_2_Se_3_ for ultralow-loss programmable silicon photonics. The favorable combination of large refractive index contrast and ultralow losses seen in Sb_2_Se_3_ facilitates an unprecedented optical phase control exceeding 10π radians in a Mach-Zehnder interferometer. To demonstrate full control over the flow of light, we introduce nanophotonic digital patterning as a previously unexplored conceptual approach with a footprint orders of magnitude smaller than state-of-the-art interferometer meshes. Our approach enables a wealth of possibilities in high-density reconfiguration of optical functionalities on silicon chip.

## Teaser

An ultralow-loss optical phase change material enables reversible programming of the flow of light in silicon photonics.

## INTRODUCTION

The birth of coherent nanophotonic processors, photonic tensor cores, quantum computing, and neuromorphic networks signifies a large paradigm shift toward emerging optical information platforms (*1*–*6*). Postfabrication programming of devices is one of the most desirable functionalities for agile reconfigurable photonic functionalities (*7*,*8*). Despite the great success of implementations based on cascaded interferometer meshes (*2*, *9*–*12*), there are inherent strong limitations in footprint scalability and volatility. Therefore, the development of new concepts and technologies is of extreme interest.

The fundamental benefits of using nonvolatile phase change materials (PCMs) in reconfigurable photonics have resulted in their extensive exploration for photonic modulation and resonance tuning, with Ge_2_Sb_2_Te_5_ (GST) (*13*–*15*) and, more recently, Ge_2_Sb_2_Se_4_Te_1_ (GSST) (*16*) being the most considered materials. The multiple nonvolatile states these materials offer provide unparalleled energy per bit operation in a highly stable platform. Commercial GST phase change memory has been shown to be stable over 10^12^ write cycles (*17*). Both materials operate based on a large change in complex refractive index ñ = *n* + i*k* between their crystalline and amorphous phases. Despite improvements in materials design, the absorption losses in one or both phases of current PCMs prevent optical phase control independent of changes in the amplitude of propagated light in the telecommunication band, severely limiting phase modulation schemes.

In several recent studies, the antimony-based chalcogenides Sb_2_S_3_ (*18*, *19*, *20*) and Sb_2_Se_3_ (*21*) have been identified as a family of highly promising ultralow-loss PCMs for photonic applications. The materials exhibit no intrinsic absorption losses (*k* < 10^−5^) in either phase over the telecommunications transmission band and show low switching temperatures around 200°C for Sb_2_Se_3_ and 270°C for Sb_2_S_3_ (*21*) while remaining nonvolatile at operating temperatures. The transition between crystalline and amorphous phases changes the arrangement of the chemical bonds in the material, which in effect results in the change of optical properties such as the complex index of refraction. Furthermore, the proximity of their refractive index to that of silicon allows straightforward direct integration of PCM patches onto standard silicon-on-insulator (SOI) integrated photonic platforms with excellent mode matching to the SOI waveguide.

Here, we demonstrate the exceptional capabilities of the PCM Sb_2_Se_3_ for use in ultralow-loss optical phase control of photonic integrated circuits (PICs). To achieve this, we make use of 23-nm-thin patches of Sb_2_Se_3_ deposited on top of a 220-nm SOI rib waveguide, where the thickness of materials is chosen to maintain a single mode of propagation. An optical phase shift of the propagating wave is induced by changing the material of the PCM from a crystalline state to an amorphous state. In our studies, following the example of optical storage media, we start from a fully crystallized PCM as this provides a uniform background, fast writing speeds, and stable amorphous written areas. Switching of individual pixels in the PCM is achieved using tightly focused optical pulses from a diode laser operating at 638-nm wavelength (see Materials and Methods).

Sb_2_Se_3_ is an emerging new material in the domain of PCMs with fundamental advantages for integrated photonics over established PCMs. To validate our approach and quantify the optical phase shifts induced by switching this PCM, we start our investigation by characterizing its response in a standard Mach-Zehnder interferometer (MZI) phase shifter configuration. The integration of Sb_2_Se_3_ PCM patches in a standard MZI is of key importance for conventional reconfigurable platforms (*5*–*10*) as it will allow replacing volatile active phase shifters with nonvolatile optical phase control.

**Fig. 1:**
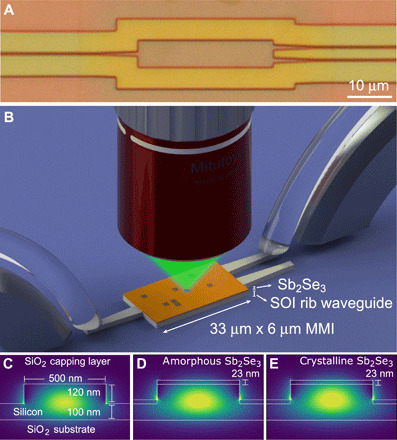
Scheme showing basic principle of optically programmable silicon photonic circuits. (**A**) Top view image of silicon photonic MMI. Scale bar, 10 μm. (**B**) Illustration of MMI with thin Sb_2_Se_3_ PCM patch and optical writing of patterns of pixels onto the device using a microscope. (**C** to **E**) Illustration of the geometry of 500-nm-wide silicon rib waveguides used in this work, without PCM patch (C) and with Sb_2_Se_3_ patch in amorphous (D) and crystalline (E) state. Simulations of the mode profile in the three cases are shown by the color map. Corresponding insertion losses between waveguide sections are shown in table S1. Photo credit: Ioannis Zeimpekis, University of Southampton.

Following the precise characterization of the optical phase shift associated with the PCM pixels in the MZI, we demonstrate the proof of principle of a programmable multiport router based on writing patterns of weakly scattering perturbations onto a multimode interference (MMI) device, such as the one shown in Fig. 1A. Figure 1B shows a schematic layout of this experiment and highlights our general approach of optical writing of pixels using a microscope. Following on earlier proof-of-principle studies (*22*, *23*), the capability of writing nonvolatile pixel patterns in a multiport waveguide signifies a breakthrough in free-form control over the flow of light inside integrated photonic circuits. Currently dense meshes of single-mode devices are being considered as the main strategy for programmable optical circuits; however, this is expensive in both the number of components and device footprint. The results presented here are a first step toward a new family of programmable multiport elements, which could open up entirely new architectures for photonic integrated circuits (*23*).

## RESULTS

### Switching of Sb_2_Se_3_ patch on a Mach-Zehnder interferometer

In our work, we use a standard 220-nm-thick SOI platform where 120-nm-thick rib waveguides are produced by partial etching of the silicon layer. The refractive index values of Sb_2_Se_3_ in its two states (*n*_amorph_ = 3.285 and *n*_cryst_ = 4.050, respectively, for amorphous and crystalline states) are close to that of silicon (*n*_Si_ = 3.48), resulting in very similar mode profiles and hence low insertion losses between the different types of waveguide configurations (see table S1). Figure 1 (C to E) illustrates the geometry of the silicon photonic platform under study and includes the mode profiles for the different configurations. From mode calculations, we found that an amorphous single pixel in an otherwise crystalline Sb_2_Se_3_ layer changes the effective refractive index of the waveguide, *n*_eff_, by an amount Δ*n*_eff_ = −0.072 (*21*).

**Fig. 2:**
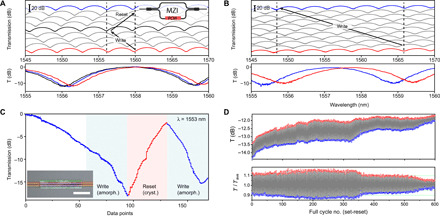
Tuning of an MZI by switching of an ultralow-loss PCM for selective optical phase control. (**A** and **B**) Spectral response of an MZI during phase tuning using laser annealing of a cladding PCM from the initial state (blue) through intermediate states (gray and black) to the final spectrum (red), vertically offset (top), and normalized to the initial state (bottom). A reversible 2π phase shift (A) and 10π tuning (B) with no change to modulation depth or insertion loss. (**C**) Transmission of an MZI for a fixed wavelength of 1553 nm, as a 50-μm patch is amorphized (blue) and recrystallized over 20 μm (red), followed by a second amorphization (blue), step size 500 nm per data point. Inset: Optical microscope image of the Sb_2_Se_3_ patch. Scale bar, 20 μm. (**D**) Transmission through an MZI as a single spot is amorphized and recrystallized 600 times (top). The same data with the drift normalized using a 50-point moving average (bottom). Photo credit: Ioannis Zeimpekis, University of Southampton.

By selective switching of the PCM in one arm of an asymmetric MZI, the optical phase shift is translated into a change of the transmission function of the device. The first demonstration is shown in Fig. 2A, in which we achieve optical phase tuning of an MZI with a 125-μm-long patch of Sb_2_Se_3_. Progressive tuning of the optical phase is achieved by amorphizing a series of spots in a continuous line along the PCM patch. Spectra were taken every 25 pixels, where each pixel is spaced by 1000 nm along the MZI. The figure shows a succession of vertically offset recorded spectra (gray) from the initial state (red) to the fully switched state (black), followed by a reset to the final state (blue). A detailed view shown at the bottom of Fig. 2A shows the initial, set, and reset states, with the insertion loss for each spectrum normalized to the initial spectrum. The insertion loss remains unchanged during this process, highlighting the lack of any losses induced by the amorphization or recrystallization. Changes in the effective refractive index Δ*n*_eff_ along the patch are expected to result in very small reflective losses of <0.02 dB per interface (see table S1). A reversible 2π phase shift was obtained as a result of 100 separate crystallization/amorphization pulses. Regardless of the small imperfections introduced by the motorized stage (backlash) during recrystallization, the full 2π range was set and reset with a 0.1-nm offset, which results in an accumulated error within 2.5% of the full range. This measurement demonstrated that a 750-nm pixel size results in a resolution of 0.02π shift, allowing very fine phase control.

As the induced optical phase change scales linearly with amorphized distance and given the ultralow loss of the material, very large phase tuning ranges can be readily achieved by using longer PCM structures. Figure 2B shows a 10π phase shift obtained by switching a 250-μm length of PCM patch, with no measurable change to insertion loss or modulation depth. The effect of the PCM on insertion loss and extinction of the MZIs is explored in more detail in fig. S2, which shows that for PCM patch lengths of up to 250 μm, no additional rebalancing of the MZI is required to compensate for losses in the PCM. Apart from using the focused laser spot size for calibration of the switched pixel, the induced optical phase can be fine-tuned by small changes in the laser power. The phase shift was tuned to 0.04π per pixel in the experiment of Fig. 2B by a small increase in optical peak power of the diode write laser. This average phase shift per pixel was calculated as the total phase shift divided by the number of pixels. Total phase shift was obtained from the spectral shift of the fringes in the MZI response.

To complement the broadband measurements, we performed additional narrowband measurements on an MZI with a shorter, 50-μm patch length as shown in Fig. 2C. Starting at the wavelength of 1553 nm, we tuned the MZI from the maximum transmission (set to 0 dB) to the minimum (−17 dB) and subsequently reset part of this device by recrystallization of a 20-μm length of the PCM patch (red curve). The curve shows the individual levels in transmission induced by switching subsequent pixels in steps of 500 nm along the PCM. An asymmetry between the first write and reset scans is observed, which is attributed to a burn-in effect of the initial film. A second amorphization scan (second blue area) follows more closely the reset scan, where we notice a small but noticeable drop in the maximum extinction of the device from −18 to −15 dB, indicative of small changes in the material upon repeated switching of the PCM layer, changing the sensitive balance between the MZI arms (see also fig. S2). Overall, this result highlights the ability to define stable multilevel switching processes for highly dense and accurate optical phase control with very fine quantization.

Next, the endurance of the phase change was probed by repeatedly switching a single pixel of the MZI. We used a pixel at the −13.5-dB point of the MZI response curve on the steepest part of the slope, where the sensitivity to individual pixels is high. By repeatedly cycling between the amorphous and crystalline phases in a single spot, the transmission modulation gives a good indication of the lifetime of the phase change. Figure 2D shows the transmission in each phase for the first 600 switches, with red dots corresponding to the crystalline and blue dots to the amorphous material phase. A long-term drift of the device transmission, due to environmental fluctuations and material changes, was observed, and we used a 50-point moving average to normalize the drift, as shown in the bottom panel of Fig. 2D. The first 350 full set-reset cycles resulted in a constant transmission change, with a small increase seen toward the 100th cycle. This increase was observed in most tested devices and is attributed to the settling of the switching dynamics after the first few burn-in cycles of the PCM, resulting in a slightly increased responsivity. A reduction of the switching contrast below 50% of the initial value is seen after around 500 cycles. Another example of an endurance experiment is shown in fig. S4. We note that the endurance seen in our current work is a factor 10 lower than that observed for Sb_2_Se_3_ on planar films (*21*), which is attributed to the somewhat more challenging thermal environment of the PCM on the waveguide and may require further improvements in thermal design.

### Digital programming of MMI using precalculated pixel patterns

Having successfully demonstrated that an optical phase shift can be induced by switching thin Sb_2_Se_3_ patch on top of an MZI, we moved on to demonstrate a new approach for a programmable optical router based on an MMI patch. MMIs are of interest for their small footprint as well as for their substantially reduced sensitivity to the environment when compared to MZIs. The device principle is based on the concept of wavefront shaping, where a distribution of weak perturbation pixels is used to effectively steer the light with very low loss (*22*,*23*). The perturbation induced by switching of the PCM layer is sufficient to apply this device concept in a practical scalable device configuration. Furthermore, a high level of control is achieved, allowing us to independently set and reset pixels spaced at 800-nm pitch in complex patterns.

**Fig. 3:**
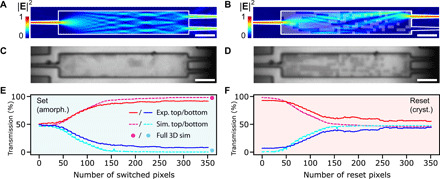
Programming of an MMI 1 × 2 splitter by writing a PCM pixel pattern. (**A** and **B**) Simulated electric field distribution for an unperturbed (A) MMI clad with 23 nm of crystalline Sb_2_Se_3_ and with a perturbed MMI with the pixel pattern of amorphous Sb_2_Se_3_ overlayed in transparent white (B). (**C** and **D**) Optical images of an experimental MMI clad with unperturbed crystalline Sb_2_Se_3_ (C) and with an amorphous pixel pattern written into Sb_2_Se_3_ (D). All scale bars, 5 μm. (**E** and **F**) Simulated transmission using 2D optimization model (green and teal dashed lines) for the top and bottom outputs of the MMI as a function of pixels written (set, amorphization) (E) and recrystallized (reset) (F) compared to the experimental values (red and blue solid lines). Dots represent final values obtained from 3D simulations with optimized pattern.

A 1 × 2 MMI device of 33 μm × 6 μm, covered with a 23-nm crystalline Sb_2_Se_3_ film, was simulated using a two-dimensional (2D) finite-difference time-domain approach, and a pixel perturbation pattern was optimized using an iterative scheme (*22*). In this scheme, we switched every pixel of the MMI individually and investigated the resulting MMI outputs. Switched pixels were kept if they improved the target response and were reset when they did not result in an improved target response. Through this iterative process, the optimum combination of pixels in the region between the input and output ports was determined, and we therefore maximized the transmission from one of the outputs. Repeating this optimization two times resulted in a pattern with high single-port transmission. Figure 3 (A and B) shows the calculated intensity in the device before and after patterning.

The designed pattern was subsequently written onto an experimental device. Figure 3 (C and D) shows the fabricated MMI with the same dimensions and cladding as the simulated ones before phase change (C) and after the perturbation pattern was written (D). A residual structure can be seen in the MMI in the unperturbed state (Fig. 3C), which is caused by the crystalline domains of Sb_2_Se_3_ in the crystalline (unswitched) state. In Fig. 3D, a very good agreement is seen in the pattern registration, with a small ~0.5° tilt present in the experimental pattern due to limitations in the alignment of the setup. Each pixel was written sequentially in columns and rows from the input to the output.

Figure 3 (E and F) shows the simulated (dashed curves) and experimental (solid lines) transmission in the top and bottom outputs for both the set (amorphization) and reset (crystallization) steps, where the single-port transmissions were obtained by normalizing to the sum of the two output ports. The absolute MMI single-port transmissions before writing are −30.6 dB (top) and −30.8 dB (bottom) compared to −25.9 dB for a straight waveguide. After writing the full pixel pattern, a 92%:8% splitting ratio was found between the top and bottom outputs, respectively. Running the final pattern from the 2D optimization sweep in a full 3D simulation for the fully patterned MMI provides a 97%:3% splitting ratio, as shown by the dots in Fig. 3E. The 3D simulation results indicate that optimization using a 2D effective-index model provides a good approximation despite the complex 3D geometry of the perturbation positioned on top of the waveguide. The 5% difference between experimental and simulated results is attributed to experimental limitations in pattern registration onto the device, which are as yet not fully understood. The device reset was done pixel-wise in the same way as the write sequence, i.e., from start to end of the pattern; therefore, the curves in Fig. 3 (E and F) are not the inverse of each other. We point out that the reset was not perfect in all areas. The thermal properties at the edges of the MMI are different due to the surrounding insulating ZnS:SiO_2_ capping layer, which results in different switching dynamics. Eventually, the material breaks down at the edges of the MMI, which results in nonreversible switching. This can be mitigated by implementing smoother interfaces and also by using a variable laser power depending on the local topology.

### Iterative optimization of MMI transmission

Next to patterning the MMI device using a calculated pixel pattern, we tested an alternative scheme of iterative optimization of the actual device. In the iterative approach, we switched individual pixels and tested their effectiveness on the target transmission. For every pixel, a test was performed to see whether switching increases the transmission at the output port. If a pixel contributed positively to the target, its switched state was preserved; otherwise, it was erased. Whereas a single-pass optimization already resulted in a notable power splitting between the two ports, additional passes are shown to further improve the target transmission function.

**Fig. 4:**
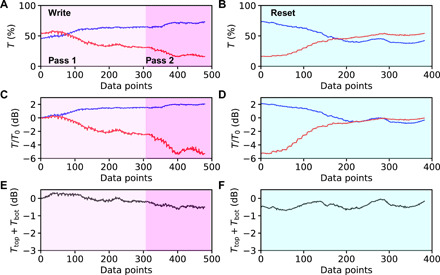
Demonstration of iterative optimization scheme. (**A** to **F**) Transmission at top and bottom MMI outputs simultaneously detected using dual-fiber output under conditions of iterative optimization (A, C, and E) and device reset (B, D, and F) at 750-nm pixel pitch. Only results for accepted pixels are shown. Panels represent raw signal intensities (A and B), log transmission normalized to the value of the unperturbed device (C and D), and the sum of top and bottom outputs normalized to the unperturbed device (E and F) showing a 0.50-dB excess loss of the patterned device. Full videos of write/reset are shown in the Supplementary Materials.

Figure 4 (A and B) shows results for bottom (blue) and top (red) output ports where only the accepted pixels are shown, and every data point corresponds to a switched or reset pixel in the MMI. Figure 4 (C and D) shows the same data on a logarithmic scale and normalized to the original port intensities of the unperturbed device, *T*_0_. Raw port output signals are subject to intrinsic variations in grating efficiency and collection fibers, therefore normalizing the signal looks at relative changes compared to the unperturbed device. The entire device was optimized in one pass (pass 1), followed by a second pass, which started again at the beginning of the device and which reoptimized the first 20 μm of the MMI (pass 2).

Figure 4C shows an increase of single-port transmission of the bottom output by 2 dB, accompanied by a 6-dB reduction of the top output. The total transmission, defined as the sum of the unnormalized *T*_top_ + *T*_bottom_, shows an overall 0.5-dB increase in insertion loss caused by writing the pattern. The changes induced by writing the pixel pattern are reversible, and the original device transmission is retrieved by resetting all pixels in the device as shown in Fig. 4 (B, D, and F). Movies of writing (both passes 1 and 2) and resetting the device are included in the Supplementary Materials. A series of three videos showing the complete iterative writing process and the device reset are presented in the Supplementary Materials. Both precalculated and iterative optimization patterns result in a target performance exceeding 8-dB extinction between the transmission from the two ports. The impact of the patterning on the total device throughput is not strongly affected, with only up to 0.5 dB additional loss for all devices under study. In some cases, an improvement in device transmission was seen, as shown for example in fig. S6. Such a small improvement in overall device throughput can be attributed to the effect of iterative wavefront shaping, which compensates for some of the imperfections of the original device, as seen in earlier studies (*22*).

### Reversibility of write and reset of MMI pixel patterns

**Fig. 5:**
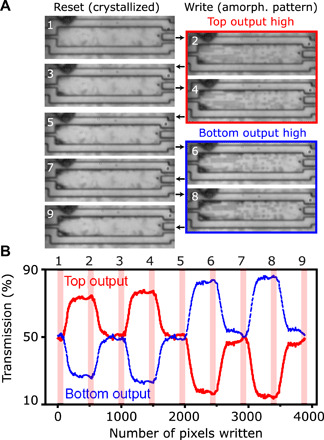
Reconfigurable optical routing between two outputs within an MMI. (**A**) Optical images of an MMI in the reset state (1, 3, 5, 7, and 9) and for patterns that are optimized for the top (2 and 4) and bottom outputs (6 and 8). Emerging dark areas at edges of device after reset (3, 5, 7, and 9) correspond to irreversible damage. (**B**) Continuous transmission ratio for the top (red) and bottom (blue) outputs of the MMI during the pattern writing and rewriting; numbers correspond to maps in (A).

To show that the pixel pattern writing approach is both reconfigurable and free-form, the same pattern was written into an MMI multiple times, with a full reset between each pattern for both the simulated pattern and the mirror image of the pattern in the vertical direction, which, due to symmetry, guides light toward the bottom output. Figure 5A shows the MMI before (1) and after (2) the first write cycle. To improve the visibility of any changes with respect to the original MMI patterns, images of all set and reset states were processed by differencing with the original MMI, as shown in Fig. 5B. A good repeatability was shown between each pattern, with the reset state showing no memory of the patterns written. After several switching cycles, incremental damage to the Sb_2_Se_3_ film occurred at the edges of the MMI. The damage appears from the corners of the device, where the lower local thermal conductivity of the surrounding ZnS:SiO_2_ capping layer and the abrupt interfaces result in a higher maximum temperature, making the Sb_2_Se_3_ film more prone to delamination or void formation. These effects can be compensated by a variety of methods such as reducing the pulse power for these areas or by reducing the actively written area of the MMI. Figure 5B shows the transmission of both outputs for all the perturbations when writing and resetting the patterns shown in Fig. 5A. A repeatable transmission change was seen for both orientations, which was not strongly affected by the increasing presence of the damaged areas (see Fig. 5A). The splitting ratio was higher for the inverse pattern, suggesting either a small misalignment between the pixel pattern and the MMI or a nonsymmetrical design due to fabrication tolerances in the Sb_2_Se_3_ film.

## DISCUSSION

The results presented here unequivocally show the capabilities of the new phase change technology in providing low-loss programmable optical phase control in PICs. This approach is expected to open many new applications in postfabrication device tuning, programmable weight banks, unitary matrix operations, and, ultimately, all-optical field-programmable arrays (*10*,*23*). Nonvolatile PCM-based approaches may hold an energy advantage compared to active devices, which require driving voltages to maintain a configuration and hence could offer opportunities for reduced power consumption PICs. The use of nonvolatile programmable MZI with large optical phase shifts exceeding 2π is of interest. Referred to as optical true time delay (OTTD), programmable optical path lengths are of interest in microwave photonics (*24*), optical fast Fourier transforms (*25*), Fourier transform sensors (*26*), and integrated quantum circuits (*27*). In microwave photonics and emerging terahertz (6G) applications (*28*,*29*), OTTDs are used for beam formation using phase array antennas and in optical communications for signal synchronization, equalization, buffering, and time division multiplexing. In quantum optics, precise tuning of optical path length differences is critical for maintaining high multiphoton coherence (*30*).

Our current results are aimed at applications in programmable and reconfigurable photonic circuits, which do not rely critically on switching speed or extreme endurance. Applications requiring extensive cycling and/or high-speed switching, such as memory cells, displays, or other adaptable elements, will require further extension of the limits of performance of these materials integrated into highly optimized device configurations. For field use, electrical switching through Joule heating may be possible. To mitigate the complexity of switching each individual pixel electrically, preselected deposited pixel arrangements could be switched with fewer connections sacrificing resolution for lower interconnect count. We envision that important aspects enabling the mass commercial use of this technology such as the reproducibility, switching endurance, and optical and electrical switching will be addressed in follow-on studies, as has been the case with decades of work in optimizing materials like GST.

In conclusion, we have demonstrated a new low loss reconfigurable approach for optical routing in an integrated silicon photonic device based on pixel patterns written on an MMI. The new PCM Sb_2_Se_3_ allows the decoupling of optical phase control from amplitude modulation seen in the conventional PCMs. The advances in device footprint and energy consumption of this approach compared to conventional cascaded switch fabrics could enable a range of complex photonic circuits needed for applications such as on-chip light detection and ranging, photonic quantum technology, artificial intelligence hardware, or optical tensor cores of the future while providing a powerful postfabrication programming technique for high-volume PIC ecosystems. Our demonstrated technique provides a general approach that could be easily extended to larger devices and could ultimately achieve a platform for a universal optical chip technology.

## MATERIALS AND METHODS

### Materials

The devices shown in this work were fabricated using an industrial deep ultraviolet (UV) scanner on a 220-nm SOI platform with a 120-nm etch depth. The UV scanner was used to open windows above the MMI and MZI regions, and 23-nm-thin Sb_2_Se_3_ films were deposited using low-temperature radio frequency (RF) sputtering. Following removal of the photoresist, the whole chip was then capped with a 200-nm-thick layer of ZnS:SiO_2_, using RF sputtering.

### Methods

A 150-mW, 638-nm wavelength single-mode diode laser was used to crystallize and amorphize Sb_2_Se_3_, using pulses of 50 ms at 19 mW and 400 ns at 35 mW, respectively. A 0.42 numerical aperture (NA) 50× objective was mounted to a 3D piezo stage, normal to the devices under test, which was for focusing the laser and taking optical images with a white light source and charge-coupled device camera. Using the piezo stage, the objective was moved to create the pixel patterns with high reproducibility. More details on the optical setup are presented in fig. S1.

## SUPPLEMENTARY MATERIALS

Supplementary material for this article is available at http://advances.sciencemag.org/cgi/content/full/7/25/eabg3500/DC1
